# Primary antiphospholipid syndrome in pediatrics: beyond thrombosis. Report of 32 cases and review of the evidence

**DOI:** 10.1186/s12969-022-00673-y

**Published:** 2022-02-14

**Authors:** Alfonso-Ragnar Torres-Jimenez, Virginia Ramirez-Nova, Adriana Ivonne Cespedes-Cruz, Berenice Sanchez-Jara, Alejandra Velazquez-Cruz, Vilma Carolina Bekker-Méndez, Francisco Xavier Guerra-Castillo

**Affiliations:** 1 Department of Pediatric Rheumatology, National Medical Center La Raza, IMSS, Vallejo y Jacarandas, colonia La Raza, Azcapotzalco, D.F. México CP, 02990 México City, México; 2 Department of Pediatric Hematology, National Medical Center La Raza, IMSS, Mexico City, México; 3 Research Unit in Immunology and Infectology, National Medical Center La Raza, IMSS, Mexico City, México

## Abstract

**Objective:**

Describe the frequency of thrombotic and non-thrombotic clinical manifestations, laboratory, treatment and prognosis in patients with pediatric primary antiphospholipid syndrome.

**Material and methods:**

A retrospective study was carried out in patients with a diagnosis of primary antiphospholipid antibody syndrome, under 16 years of age, under follow-up by the pediatric rheumatology service of the General Hospital, National Medical Center, La Raza, from January 2013 to December 2020. The antiphospholipid syndrome was defined when it met the laboratory criteria of the Sidney criteria and the presence of thrombosis or non-criteria manifestations of the disease (hematological, neurological, cutaneous, renal, cardiac or pulmonary). Demographic, clinical, laboratory, treatment, and prognosis data were collected.

**Results:**

We report 32 patients, 21 female (65%) and 11 male (35%), mean age 11.75 years, evolution time 16 weeks. Thrombosis 9 patients (28%), 1 arterial and 8 venous. Non-thrombotic manifestations; Hematologic: thrombocytopenia 22 patients (69%), autoimmune hemolytic anemia 13 (40%), Fisher-Evans syndrome 6 (19%), lupus anticoagulant with hypoprothrombinemia syndrome 2 (6%). Dermatological: livedo reticularis 20 (62%), skin ulcers 2 (6%), Raynaud's phenomenon 8 (25%). Neurological: epilepsy 1 (3%), migraine 3 (9%), chorea 1 (3%) and cognitive impairment 3 (9%). Renal in 4 (13%). Laboratory: prolonged aPTT 30 (93%), lupus anticoagulant 32 (100%), positive IgG anticardiolipin 20 (62%), positive IgM anticardiolipin 19 (60%). AntiB2GPI was performed in only 3 patients, being positive in all. Treatment: anticoagulation in patients with thrombosis, antiplatelet in 23 (72%), steroid 30 (94%), immunosuppressant 30 (94%) and rituximab 4 (12.5%). No deaths were reported.

**Conclusions:**

The clinical characteristics of patients with pediatric primary antiphospholipid syndrome differ from those presented in adults, since non-thrombotic manifestations are more frequent in children, for which classification criteria that include these manifestations are necessary for a better characterization of the disease in pediatric population.

## Introduction

Antiphospholipid syndrome is an acquired immune mediated prothrombotic state. It is an important cause of thrombosis and obstetric loss. The history of this condition can be traced back to observations made during syphilis detection programs carried out in the first half of the twentieth century with the use of purified cardiolipin mainly in the Venereal Disease Research Laboratory (VDRL) microflocculation assay. In 1952 researchers from Johns Hopkins University reported an approximate incidence of false positive VDRL in 20% of patients with lupus, which was later associated with the presence of lupus anticoagulant. In 1960, this in vitro anticoagulant phenomenon was associated with thrombosis rather than hemorrhage. Later in 1983, Harris used radioimmunoassay to show that at least two-thirds of serum samples from a group of 65 lupus patients had elevated levels of anticardiolipin antibodies. In addition, 90% of these patients who had lupus anticoagulant positivity had elevated levels of anticardiolipin antibodies [1, 2].

It was in 1987 when the Hammersmith hospital group proposed the first criteria for this syndrome, which included three clinical criteria (arterial or venous thrombosis, obstetric loss and thrombocytopenia) and two laboratory criteria (LAC and ACL) [3, 4]. In subsequent years, several authors began to describe other manifestations associated with this syndrome such as chorea, livedo reticularis, autoimmune hemolytic anemia, transverse myelitis, migraine, epilepsy, Raynaud's phenomenon, and cardiac valve lesions. In the late 1980s and early 1990s, the term primary antiphospholipid syndrome was used when it was not associated with another autoimmune disease. [3–10]. In 1992, other criteria were described for the classification of the antiphospholipid syndrome that complicates lupus, considering that to be classified as an antiphospholipid syndrome, they should meet two clinical criteria (livedo reticularis, recurrent obstetric loss, venous thrombosis, thrombocytopenia, hemolytic anemia, arterial thrombosis, skin ulcers) plus elevated levels of antiphospholipid antibodies [11].

It was not until 1998 when the 8th International Symposium on Antiphospholipid Antibodies was held in Sapporo, Japan, addressed the need to unify criteria for classification, define the essential characteristics of the syndrome and facilitate treatment and etiology studies and defined the clinical manifestations and laboratory tests closely related to antiphospholipid syndrome. In this consensus, vascular thrombosis and obstetric morbidity remained as clinical criteria, and the presence of LAC and ACL dependent on B2GPI as laboratory criteria. Other clinical criteria (thrombocytopenia, autoimmune hemolytic anemia, transverse myelitis, livedo reticularis, heart valve disease, chorea and migraine) were excluded as it was considered that there was no strong evidence based on clinical or experimental investigations [12]. In 2004, the criteria were revised in Sydney, adding anti-B2GPI antibodies to the laboratory criteria [13]. These criteria was designed for non clinical (research) purposes and are a useful tool to limit the overdiagnosis of antiphospholipid syndrome; however, they do not encompass all clinical manifestations, so the final decision is always based on the judgment of the treating physician [14].

At this point, it must be considered that these criteria were based on studies carried out in adults and that in children obstetric morbidity is rare, and the risk factors for thrombosis are lower than those presented in adults, so it is clear to assume that these manifestations are less frequent in children, and that diagnostic criteria focused on pediatric patients are needed. Therefore, the antiphospholipid syndrome is not well defined in children and there are no validated criteria for this age. The criteria for adults are specific but lack sensitivity when applied to children, so the incorporation of non-criteria clinical manifestations is important in the pediatric population [15].

Thus, prothrombotic risk factors such as age, smoking, obesity, hypertension, dyslipidemia, diabetes, use of oral contraceptives, pregnancy, congestive heart failure, varicose veins and cancer are more frequent in adults [16–18]. In contrast to these, prothrombotic risk factors in children are trauma, surgery, neoplasm, nephrotic syndrome, congenital heart disease, obesity, central venous catheters, prolonged immobilization, stay in the ICU, burns and mechanical ventilation [19, 20], which are less prevalent in this population, and might explain the lower number of thrombotic events in pediatric antiphospholipid syndrome.

## Material and methods

A retrospective study was carried out in patients with a diagnosis of primary antiphospholipid antibody syndrome, under 16 years of age, followed by the pediatric rheumatology service of the General Hospital, National Medical Center, La Raza, Mexico, from January 2013 to December 2020. Pediatric primary antiphospholipid syndrome (PAPS) was defined when the child fulfilled the laboratory criteria of the Sidney criteria on ≥ 2 occasions at least 12 weeks apart and presented thrombosis or non-criteria manifestations of the disease (hematological, neurological, cutaneous, renal, cardiac or pulmonary). Demographic, clinical, laboratory, treatment, and prognosis data were collected. Patients who did not meet Sidney's laboratory criteria, older than 16 years and who had another associated rheumatological disease, were excluded.

Descriptive statistics with frequencies and percentages were used for categorical variables, in continuous variables with mean, minimum and maximum. Associations between categorical variables were measured with χ2, and for continuous variables with Mann-Whitney U test or T test, using the SPSS 25 statistical software. Differences were considered statistically significant at *P* < 0.05.

## Results

We present the results of 32 patients, 21 female (65%) and 11 male (35%). The average age at diagnosis 11.75 years (1–15 years): 12.2 years (1–15 years) in those with thrombosis, and 11.5 years (6 to 15 years) in those with only non-thrombotic manifestations. The average evolution time from the onset of symptoms was 16 weeks (1–108 weeks) in all patients: 7.5 weeks (1–28 weeks) in patients with thrombosis and 19 weeks (1–108 weeks) in patients without thrombosis. Thrombosis occurred in 9 patients (28%), 1 arterial and 8 venous, one site involving in 6 patients and 2 or more sites in 3. The most frequent site of venous thrombosis included the femoral vein and in the patient with arterial involvement it was cerebral.

Regarding non-thrombotic manifestations, hematological involvement was thrombocytopenia in 22 patients (69%), mean platelets with 119,000 (1,000–399,000) in all patients, but 54,000 (1,000–145,000) in patients with thrombocytopenia, autoimmune hemolytic anemia in 13 (40%), Fisher Evans syndrome in 6 (19%), leukopenia (< 4500) in 4 (12.5%), lymphopenia (< 1500) in 6, bleeding or ecchymosis in 16 (50%). The anemia was due to cold antibodies in 10, warm in 2 and mixed in 1. Lupus anticoagulant with hypoprothrombinemia syndrome was presented in 2 (6%). The dermatological manifestations were livedo reticularis in 20 (62%), skin ulcers in 2 (6%), and Raynaud's phenomenon in 8 (25%). Neurological manifestations include: epilepsy 1 (3%), migraine 3 (9%), chorea 1 (3%) and cognitive impairment 3 (9.4%). No cardiopulmonary manifestations were observed. Renal involvement was found in 4 (13%) (nephrotic syndrome 1 and nephritic syndrome 3). (Table 1).

**Table 1:** Demographic and clinical characteristics

	All patients *n* = 32 (%)	Thrombosis *n* = 9 (%)	Non-thrombotic *n* = 23 (%)	*P* value
Sex (female/male)	21/11	4/5	17/6	0.11
Age (years)	11.7 (1–15)	12.2 (1–15)	11.5 (6–15)	0.93
Evolution time (weeks)	16 (1–108)	7.5 (1–28)	19 (1–108)	0.21
Hematologic	29 (90%)	7 (78%)	22 (96%)	
Thrombocytopenia	22 (67%)	7 (78%)	15 (65%)	0.49
AIHA	13 (41%)	2 (22%)	11 (48%)	0.18
LAHP	2 (6%)	0	2 (9%)	0.36
Dermatologic	23 (72%)	7 (78%)	16 (70%)	
Livedo reticularis	20 (63%)	7 (78%)	13 (57%)	0.26
Raynaud's phenomenon	8 (25%)	2 (22%)	6 (26%)	0.82
Skin ulcers	2 (6%)	0	2 (9%)	0.36
Neurologic	5 (16%)	1 (11%)	4 (17%)	
Migraine	3 (9%)	1 (11%)	2 (9%)	0.83
Cognitive impairment	3 (9%)	1 (11%)	2 (9%)	0.83
Epilepsy	1 (3%)	1 (11%)	0	0.10
Chorea	1 (3%)	0	1 (4%)	0.52
Renal	4 (13%)	3 (33%)	1 (4%)	
Nephrotic síndrome	1 (3%)	1 (11%)	0	0.10
Nephritic syndrome	3 (9%)	2 (22%)	1 (4%)	0.11
Clinical profile				
T	1 (3%)	1 (11%)	0	
H	6 (19%)	0	6 (26%)	
R	1 (3%)	1 (11%)	0	
C	1 (3%)	0	1 (4%)	
H + C	16 (50%)	5 (55%)	11 (48%)	
H + N	1 (3%)	0	1 (4%)	
H + C + R	2 (6%)	1 (11%)	1 (4%)	
H + C + N	3 (9%)	0	3 (13%)	
H + C + N + R	1 (3%)	1 (11%)	0	

*LAHP* Lupus anticoagulant with hypoprothrombinemia syndrome, *AIHA* Autoimmune hemolytic anemia, *T* Thrombosis, *H* Hematologic, *R* Renal, *C* Cutaneous, *N* Neurologic

Exclusively non-thrombotic manifestations were observed in 23 patients (72%); only thrombotic in 1 (3%); thrombotic and non-thrombotic in 8 (25%). Among all patients with non-thrombotic manifestations, the combination of hematological (thrombocytopenia, autoimmune hemolytic anemia or lupus anticoagulant with hypoprothrombinemia syndrome) and cutaneous (livedo reticuaris, Raynaud's phenomenon, skin ulcers) was found in 16 (51%), hematological, cutaneous and renal (nephrotic or nephritic syndrome) in 2 (6%), hematological, cutaneous, renal and neurological (chorea, migraine, epilepsy) in 1 (3%), hematological, cutaneous and neurological in 3 (9%), hematological and neurological in 1 (3%), only hematological in 6 (19%), only renal in 1 (3%) and only cutaneous in 1 (3%). The majority of the patients (*n* = 22) presented hematological and cutaneous manifestations plus some other manifestation. The differences between the profile of clinical characteristics presented in patients with thrombosis and non-thrombotic manifestations are shown in Table 1.

In laboratory tes ts, prolonged aPTT was observed in 30 patients (93%), average of 77 s (27–124 s, normal value 30 s), a lupus anticoagulant in 32 (100%), average value 2 (1.22- 3.39, normal value 1.2), anticardiolipin IgG antibodies in 20 (62%), mean value of 191 GPL (47–280 GPL), anticardiolipin IgM antibodies in 19 (60%), mean value of 153 MPL (41–255 MPL). AntiB2GPI was performed in only 3 patients, being positive in all.

Regarding the presence of laboratory abnormalities supporting APS, two subjects had only the lupus anticoagulant, 10 subjects had both LAC and IgG anticardiolipin antibody, 8 had LAC with IgM ACL antibodies, and 9 had LAC with both IgM and IgG ACL antibodies. Of the three children with antii-B2GPI antibodies, 2 had LAC, ACL-IgM antibodies and anti-B2GPI IgG, and 1 had LAC, IgG ACL and anti-B2GPI IgG antibody. The differences between the laboratory profile presented in patients with thrombosis and non-thrombotic manifestations are shown in Table 2.

**Table 2:** Laboratory and treatment characteristics

	All patients *n* = 32 (%)	Thrombosis *n* = 9 (%)	Non-trombotic *n* = 23 (%)	*P* value
Prolonged aPTT	30 (94%)	9 (100%)	21 (91%)	0.36
Value in seconds (NV = 33 s)	77 (27-124 s)	97 (41-124 s)	69 (27–120)	0.033*
Lupus anticoagulant (dRVVT)	32 (100%)	9 (100%)	23 (100%)	
Value (NV = < 1.2)	2.07	2.13	2.05	0.71
Anticardiolipin antibodies	29 (91%)	8 (88%)	21 (91%)	
IgG	20 (63%)	6 (66%)	14 (61%)	0.76
Value (NV < 40)	191 GPL	188 GPL	192 GPL	0.86
IgM	19 (59%)	4 (44%)	15 (65%)	0.28
Value (NV < 40)	153 MPL	154 MPL	153 MPL	0.50
Antibody profile				
LAC	2 (6%)	1 (11%)	1 (4%)	0.47
LAC + ACL IgG	10 (31%)	4 (44%)	6 (26%)	0.31
LAC + ACL IgM	8 (25%)	0	8 (35%)	0.041*
LAC + ACL IgG + IgM	9 (28%)	2 (22%)	7 (30%)	0.64
LAC + ACL IgM + B2GPI	2 (6%)	2 (22%)	0	
LAC + ACL IgG + B2GPI	1 (3%)	0	1 (4%)	
Treatment				
Anticoagulation	9 (28%)	9 (100%)	0	
Antiplatelet	23 (72%)	0	23 (100%)	
Steroid	30 (94%)	8 (88%)	22 (96%)	
Immunosuppressant	30 (94%)	8 (88%)	22 (96%)	
Cyclophosphamide	8 (25%)	6 (66%)	2 (9%)	
Mycophenolate	18 (56%)	1 (11%)	17 (74%)	
Azathioprine	4 (13%)	1 (11%)	3 (13%)	
Rituximab	4 (13%)	2 (22%)	2 (9%)	

*aPTT* Activated partial thromboplastin time, *LAC* Lupus anticoagulant, *ACL* Anticardiolipin antibodies, *NV* Normal value, *dRVVT* Diluted Russell Viper Venom Time

All patients of the 9 with thrombosis received anticoagulation (warfarin in 1, acenocoumarin in 3 and enoxaparin in 5). 23 (72%) received anti-platelet medication. 30 of 32 (94%) patients received glucocorticoid therapy, and 94% received immunosuppressant treatment (8 cyclophosphamide, 18 mycophenolate, and 4 azathioprine). Four (12.5%) received rituximab.

A difference was found between the aPTT value and the presence of thrombosis (97 vs 69 s *p* = 0.035). The anticardiolipin IgG value was lower in patients with autoimmune hemolytic anemia (68 vs 165, *p* = 0.022). IgM anticardiolipin values were significantly higher in patients with autoimmune hemolytic anemia (139 vs 66 *p* = 0.009). If the patient have a positive IgM ACL, a correlation was found with autoimmune hemolytic anemia (*p* = 0.016, RR 2.1, 95% CI 1.1–3.5). The antibody profile that was related to non-thrombotic manifestations was LAC + ACL IGM (*p* = 0.041 RR 1.53 95% CI 1.13–2.06). The presence of bleeding was higher in patients with a lower number of platelets (72,000 vs. 165,000 *p* = 0.007). There were no deaths in our patients.

## Discussion

### Epidemiology

There are no reliable data on the incidence and prevalence of pediatric PAPS, given the lack of validated criteria; however, of all cases of antiphospholipid syndrome in pediatric age, it is estimated that 24–50% are primary. The average age of presentation is from 10.7 to 14 years, but it can occur from the neonatal period to adolescence, with a male:female ratio of 1: 1.2. Up to 21% progress to SLE in 6 years [21–26]. The age of presentation coincides with our results, the male: female ratio being a little higher at 1: 1.9 in our cohort.

### Clinical manifestations

#### Thrombosis

In a study carried out by Avcin in 2008, in 121 pediatric patients with APS from 24 centers in 14 countries, it was found that 60 patients (49.5%) had PAPS, presenting with venous thrombosis in 60% (most frequently venous thrombosis of the lower extremities), arterial thrombosis in 32%, (more frequent ischemic (CVD) cerebrovascular disease), small vessel thrombosis (digital ischemia) in 6% and mixed in 2%. [27] Jingran reported a study of 58 patients with pediatric APS, finding venous thrombosis in 53% (deep vein thrombosis and pulmonary embolism) and arterial thrombosis in 21% [28]. Of these patients, 24% had PAPS. Amaluya reported 17 patients in a 20-year period at the Mayo Clinic in whom there was arterial thrombosis occurred in 35% and venous thrombosis in 65% [29]. In our study, venous thrombosis of the lower limbs was more frequent, which occurred in 89% of the patients who presented with thrombosis and only 1 patient with arterial thrombosis (11%).

An interesting study presented by Avcin at the 20th Pediatric Rheumatology European Society (PReS) Congress in 2013, followed 159 patients under 18 years of age with at least one positive antiphospholipid antibody for 6 years, of which only 25 (16%) presented thrombosis. (16 venous and 9 arterial), contrasting with other non-thrombotic manifestations such as hematological (30%), non-thrombotic neurological (16%) and cutaneous (3%), showing that thrombosis does not always develop in pediatric patients with positive antiphospholipid antibodies. [30] These results are different from our study since non-thrombotic manifestations were more frequent (97%) and thrombosis only occurred in 28%. In our 9 patients who presented with thrombosis, it was associated with hematological and skin manifestations in 7 (78%).

### Non-criteria manifestations

#### Hematological

Thrombocytopenia is one of the main laboratory manifestations in pediatric PAPS, occurring in 8 to 38% of patients. It is usually moderate (> 50,000), bleeding is infrequent, and usually presents as petechiae or ecchymosis. The cause of thrombocytopenia is believed to be the direct binding of antiphospholipid antibodies to platelet phospholipids, antibodies to platelet glycoproteins, or platelet activation. Regarding autoimmune hemolytic anemia, it occurs in 6 to 21% of patients and has been associated with anticardiolipin positivity, heart valve disease and livedo reticularis and is due to a cross-reaction of antiphospholipids with phospholipids of the erythrocyte membrane (phosphatidylcholine). The combination of autoimmune hemolytic anemia with thrombocytopenia (Fisher Evans syndrome) occurs in 10% to 15% and is associated with very high levels of IgG and IgM anticardiolipins. Other manifestations that may occur but are very rare, are bone marrow necrosis and pure red series aplasia. [27, 28, 31, 32]. Another rare but life-threatening manifestation is the lupus anticoagulant with hypoprothrombinemia syndrome, which presents with prolonged PT and aPTT, positive lupus anticoagulant, and decreased factor II, due to anti-prothrombin antibodies that increase the clearance of factor II. The clinical manifestations in 74 patients with this complication were hemorrhage in 89% (51% severe, mainly gynecological) and thrombosis in 13% [33, 34]. The frequency of Fisher Evans syndrome coincides with that reported in the literature (19%). In our cohort of patients, 29 (90%) presented some hematological manifestation (thrombocytopenia, autoimmune hemolytic anemia or lupus anticoagulant syndrome with hypoprothrombinemia), being higher than that reported in the literature, but our study included patients with non-thrombotic manifestations, which likely explains the difference.

### Dermatological manifestations

Skin manifestations occur in 18 to 26% of pediatric PAPS patients and though none are pathognomonic, livedo reticularis and Raynaud's phenomenon being the most frequent and digital gangrene and skin ulcers less frequent [27, 28, 35]. In our patients, 71% presented with skin manifestations (livedo reticularis, Raynaud's phenomenon or skin ulcers), being greater than that reported in the literature.

### Neurological manifestations

Neurological manifestations in antiphospholipid syndrome can be related to thrombosis, inflammation, and direct effects of APL antibodies on neuronal function. Non-thrombotic neurologic manifestations occur in 12–16% of patients and include migraine headache, chorea, epilepsy, pseudotumor cerebri, and conduct disorders [27, 36]. Headache is the most common symptom (20%), but large studies have failed to establish a relationship with APS. Nevertheless, some patients respond to anticoagulation management. Seizures occur in 3–8% and the pathogenesis may be related to microthrombosis or immune-mediated neuronal damage. Seizures are associated with the presence of thrombocytopenia and livedo reticularis. Chorea is rare and occurs in 1.3 to 4.5%. Chorea can be generalized or unilateral and is due to the effect of antiphospholipid antibodies on the basal ganglia. Other manifestations that can be observed are multiple sclerosis like, transverse myelitis, psychosis, and Guillian Barre-like syndrome [36–39] In our study, we found neurological manifestations in 16% (chorea, epilepsy, migraine and cognitive defects), which is consistent with the literature. It is important to mention that in our study, at least two neurological manifestations were found in each patient with neurologic involvement.

### Cardiopulmonary manifestations

Heart valve abnormalities are seen in 40 to 60% of adults but are rarely seen in pediatric patients; other manifestations are coronary occlusive disease, cardiomyopathy, and intracardiac thrombosis [40, 41]. Pulmonary manifestations mainly comprise pulmonary thromboembolism, and rarely pulmonary hypertension, alveolar hemorrhage, fibrosing alveolitis and pulmonary infarction [42, 43]. In our study, thrombotic or non-thrombotic cardiac or pulmonary manifestations were not observed, which contrasts with the data obtained in the adult population.

### Renal manifestations

Thrombotic renal manifestations occur in 1 to 3%, include thrombotic microangiopathy, renal vein thrombosis, and renal artery thrombosis or stenosis, however, not only renovascular involvement is observed, since minimal change disease, membranous, proliferative glomerulonephritis and mesangial nephropathy may also occur [27, 44–46]. We did not observe cases of thrombotic microangiopathy, only 3 patients with nephritic syndrome and one with nephrotic syndrome, the latter associated with renal vein thrombosis. Kidney biopsy was not performed in any patient.

### Laboratory

In the adult APS classification criteria, persistent lupus anticoagulant, anticardiolipin and anti-B2GPI antibodies are considered, requiring they be positive for at least 12 weeks. It is recommended to perform the three tests (LAC, ACL and anti B2GPI) to stratify the risk of thrombosis. In patients with pediatric PAPS, the presence of anticardiolipin was found in 82% (IgG 37%, IgM 22%, both 28%), anti B2GPI in 70% (IgG 35%, IgM 17%, both 17%) and lupus anticoagulant in 72% [27, 47]. In our study, lupus anticoagulant was found in 100%, positive anticardiolipin in 91% (IgG 33%, IgM 31%, both 27%) being similar than previously reported. Only 2 patients had abnormal results in a single test (LAC), and the rest of the patients had 2 or 3 positive tests. Unfortunately, in our institution, the determination of antiB2GPI is not routinely utilized, so it was only performed in 3 patients, in all of whom, it was positive. No antibody profile was associated with the presence of thrombosis in our study.

### Treatment

In 2017, the SHARE initiative issued recommendations for the treatment of pediatric APS, focusing on the thrombotic manifestation. These include the use of antimalarials, antiplatelet agents and anticoagulants [15].

For non-thrombotic manifestations, multiple treatments have been used depending on the type of manifestation, such as glucocorticoids and immunosuppressants (IVIG, azathioprine, mycophenolate, cyclosporine, cyclophosphamide, plasmapheresis and rituximab), as well as symptomatic treatments when necessary (antiepileptics, antihypertensives, vasodilators). In our study, since most of the patients presented 2 to 3 clinical manifestations, the use of steroids and immunosuppressants occurred in 30 patients (94%). Rituximab was used in 4 patients (2 with thrombocytopenia, 1 with thrombocytopenia, thrombosis, neurological and kidney disease, and 1 with thrombosis and kidney disease).

### Classification criteria

There are no validated criteria for the diagnosis of pediatric PAPS. The Sydney criteria were adapted for the pediatric population and excluded obstetric morbidity, however these criteria are only used for research purposes to homogenize populations [48]. For clinical and diagnostic purposes, they are not useful, since as we demonstrated previously, the clinical manifestations of pediatric patients with persistently positive antiphospholipid antibodies go beyond thrombosis. In fact, non-thrombotic manifestations are even more frequent than thromboses in children. Therefore we suggest incorporating these non-thrombotic APS manifestations (hematological, cutaneous, neurological and renal) to the criteria in order to allow earlier diagnosis, prevent damage, and improve their quality of life with adequate treatment.

Efforts have been made to include new manifestations in the criteria for the classification of antiphospholipid syndrome, including cardiac, hematological, macrovascular, microvascular and neurological manifestations, however, clinical studies are still needed to validate them and their focus is not on pediatric patients. [49, 50].

## Conclusions

Antiphospholipid syndrome in the pediatric age group cannot continue to be studied as in adult patients, since the characteristics of this population are different, considering that thrombotic and obstetric manifestations are less frequent at this age, given the absence of obstetric morbidity and the lower frequency of prothrombotic risk factors, making non-thrombotic manifestations of the disease more evident, such as those mentioned in this study. There is a need to redefine this syndrome in the pediatric population as mentioned above and to generate its own classification criteria, in order to improve epidemiological, clinical and treatment studies of this type of patients.

## Data Availability

The datasets used during the current study are available from the corresponding author on reasonable request.

## Abbreviations

aPTT: Activated partial thromboplastin time
VDRL: Venereal disease research laboratory microflocculation assay
LAC: Lupus anticoagulant
ACL: Anticardiolipin antibodies
ICU: Intensive care unit
SLE: Systemic lupus erythematosus
PAPS: Primary antiphospholipid syndrome
CVD: Cerebrovascular disease
APS: Antiphospholipid síndrome
SHARE: Single Hub and Access point for paediatric Rheumatology in Europe
IVIG: Intravenous immunoglobulin
LAHP: Lupus anticoagulant with hypoprothrombinemia syndrome
AIHA: Autoimmune hemolytic anemia
